# Spatial transcriptomics unveils estrogen-modulated immune responses and structural alterations in the ectocervical mucosa of depot medroxyprogesterone acetate users

**DOI:** 10.1038/s41598-024-83775-9

**Published:** 2025-01-06

**Authors:** Vilde Kaldhusdal, Mathias Franzen Boger, Annelie Tjernlund, Adam D. Burgener, Frideborg Bradley, Julie Lajoie, Kenneth Omollo, Joshua Kimani, Keith Fowke, Paulo Czarnewski, Kristina Broliden

**Affiliations:** 1 Department of Medicine Solna, Division of Infectious Diseases, Center for Molecular Medicine, Karolinska University Hospital, Karolinska Institutet, Bioclinicum J7:20, 171 76 Solna, Sweden; 2 Center for Global Health and Diseases, Department of Pathology, Case Western Reserve University, Cleveland, OH 44106 USA; 3 Department of Obstetrics & Gynecology, University of Manitoba, Winnipeg, MB R3E 3P5 Canada; 4 Department of Medical Microbiology and Infectious Diseases, University of Manitoba, Winnipeg, MB R3E 0J9 Canada; 5 Department of Medical Microbiology and Immunology, University of Nairobi, Kenyatta National Hospital Campus, 30197-00100, Nairobi, Kenya; 6 Partners for Health and Development in Africa, 3737-00506, Nairobi, Kenya; 7 Department of Community Health Sciences, University of Manitoba, Winnipeg, MB R3E 0W3 Canada; 8 Science for Life Laboratory, Department of Biochemistry and Biophysics, National Bioinformatics Infrastructure Sweden, Stockholm University, 171 65 Solna, Sweden

**Keywords:** DMPA, Spatial transcriptomics, Ectocervix, Hypoestrogenemia, Mucosa, Estrogen, Transcriptomics, Molecular medicine

## Abstract

The injectable contraceptive, depot medroxyprogesterone acetate (DMPA), is associated with compromised cervical mucosal barriers. High-resolution spatial transcriptomics is applied here to reveal the spatial localization of these altered molecular markers. Ectocervical tissue samples from Kenyan sex workers using DMPA, or non-hormonal contraceptives, underwent spatial transcriptomics and gene set enrichment analyses. Integrated systemic estradiol levels and bulk tissue gene expression data from a larger cohort enhanced the study’s scope. Unsupervised clustering unveiled four epithelial and seven submucosal layers, showcasing spatially restricted and diverse functional epithelial responses, and a less structured submucosal spatial ordering. DMPA associated with mucosal-wide immunoglobulin gene upregulation, verified by CD20^+^ B-cell immunostaining, and upregulated immune markers adjacent to the basal membrane. Downregulated genes represented spatially restricted disrupted epithelial barrier integrity and submucosal extracellular matrix dysfunction. The transcriptional profile was associated with markers of estrogen regulation. Collectively, our findings reveal estrogen-modulated distinct ectocervical transcriptional profiles associated with DMPA usage. While upregulation of immunoglobulin genes occurs throughout the mucosa, activation of innate immune responses and dysregulation of barrier integrity markers are spatially restricted. These results extend previous analyses using bulk transcriptomics and provide insights into the molecular landscape influenced by DMPA, shedding light on contraceptive effects and health implications.

## Introduction

Depot medroxyprogesterone acetate (DMPA) stands as a highly effective hormonal contraceptive with a user base exceeding 55 million globally. Primarily prescribed in regions coinciding with elevated human immunodeficiency virus (HIV) incidence and maternal morbidity rates^1,2^, DMPA has undergone scrutiny in observational epidemiological studies and systematic meta-reviews^3–5^. These investigations suggest an increased HIV risk among DMPA users, though dissenting perspectives exist^6^. A large randomized controlled clinical trial, the ECHO study, did not find a statistically significant difference in HIV acquisition between DMPA users and those using implants or copper intrauterine devices^7^. The trial’s conclusions have prompted concerns^8^, paralleling doubts about the validity of prior observational studies^6^.

Gene expression profiling studies of ectocervical tissues from DMPA users reveal compromised mucosal integrity and heightened inflammatory responses^9,10^. These investigations were based on bulk transcriptomics analysis of whole tissue biopsies, demonstrating similarities across diverse study designs and target populations. Other observational studies of women using DMPA showed higher abundance of a non-optimal microbiome, increased expression of inflammatory markers, increased CD4 cell density, and epithelial disruption of the cervicovaginal mucosa, whereas sub-studies of the ECHO trial found an increase in Th17-like cells, but no adverse changes in the cervicovaginal microbiome composition, inflammation, proteome, transcriptome, or risk of sexually transmitted infections^11^. Nonetheless, it remains crucial to unravel how contraceptive compounds impact the cervicovaginal mucosa, the primary portal for HIV entry in sexual transmission. Advancements in newer technology now allow for a higher spatial resolution of proposed mechanisms of mucosal remodeling, complementing previous studies using clinical, animal, and experimental models^12–17^.

The active component of DMPA exhibits affinity for both progesterone and glucocorticoid receptors, thereby impacting numerous immune functions and epithelial stability processes in the genital mucosa^17^. The interpretation of how DMPA use affects HIV susceptibility is complex due to these partly counteracting processes. Spatial transcriptomics has revolutionized the understanding of biological processes by defining the exact location and cell origin of altered gene expression in tissue^18–20^. Recently applied to the human cervical epithelium, it has unveiled spatiotemporal molecular alterations during human papilloma virus associated cancer development^21,22^.

DMPA suppresses ovulation and reduces ovarian production of estrogen by inhibiting secretion of GnRH from the hypothalamus. This, in turn, leads to reduced production of LH and FSH and a subsequent reduction of ovarian hormones, including estrogen. Ectocervical tissue samples from DMPA users have been characterized by gene expression profiling using bulk transcriptomics, thus revealing impaired cervicovaginal mucosal integrity and increased inflammatory responses^9,10^. In this study, we further extended these investigations by spatially resolving the molecular signatures, stratified across distinct layers of the ectocervical epithelium and submucosa. The hypoestrogenic response in DMPA users was implicated as a functional driver of these responses.

## Results

### Characterization of the study participants

This study incorporated ectocervical tissue samples previously obtained as part of our broader longitudinal investigation within the Sex Worker Outreach Program in Pumwani, Nairobi, Kenya^10,23^. The samples represented women who had been using DMPA for more than 6 months (DMPA group; *n* = 4), or non-hormonal contraceptives who were in the follicular menstrual stage (control group; *n* = 4) (Table 1). The sample selection criteria included RNA integrity number above 7, and an adequate tissue morphology for allowing visualization of epithelial and submucosal gene expression. The study groups were matched based on cervicovaginal microbiota composition representative of the larger study cohort, and lack of bacterial vaginosis^24^. Considering the impact of DMPA concentration on natural serum estradiol levels, we further selected two samples from the DMPA group with estradiol levels within the range of the control group and two with levels below the limit of detection, the latter representing recent DMPA injection.

**Table 1:** Sociodemographic and clinical characteristics of study participants at time of sample collection.

	DMPA (n = 4)	Controls^a^ (n = 4)
	Median or number (range)	Median or number (range)
Age (years)	28 (25–32)	30 (20–38)
Time in sex work (months)	24 (18–24)	30 (2–36)
Plasma hormone levels		
*Estradiol*^*b*^*(pg/ml)*	57 (22–124)	61 (34–104)
Below LLD^c^ (22 pg/ml)	2	0
*Progesterone (ng/ml)*	0.05 (0.05–0.05)	0.06 (0.05–9.4)
Below LLD (0.05 ng/ml)	4	2
Having a regular partner		
No	0	1
Yes	3	3
No answer	1	0
Cervicovaginal microbiota composition^d^		
L2	3	3
L3	1	1
Bacterial vaginosis (Based on nugent score)		
Normal (0–3)	4	4
Intermediate (4–6)	0	0
BV (7–10)	0	0

^a^Controls: non-hormonal contraceptive users.

^b^*Estradiol* levels:

DMPAs: P114: 92 pg/ml, P107: 124 pg/ml, P097 and P108: < 22 pg/ml.

Controls: P118: 34 pg/ml, P080: 46 pg/ml, P031: 76 pg/ml, P105: 104 pg/ml.

^c^LLD: Lower limit of detection.

^d^Cervicovaginal microbiota composition:

L2 (> 80% Lactobacillus spp.), L3 (Gardnerella > 10% and Prevotella < 5%), as defined by Edfeldt et al.^24^.

### Identification of distinct gene clusters across the ectocervical mucosal layers

**Fig. 1:**
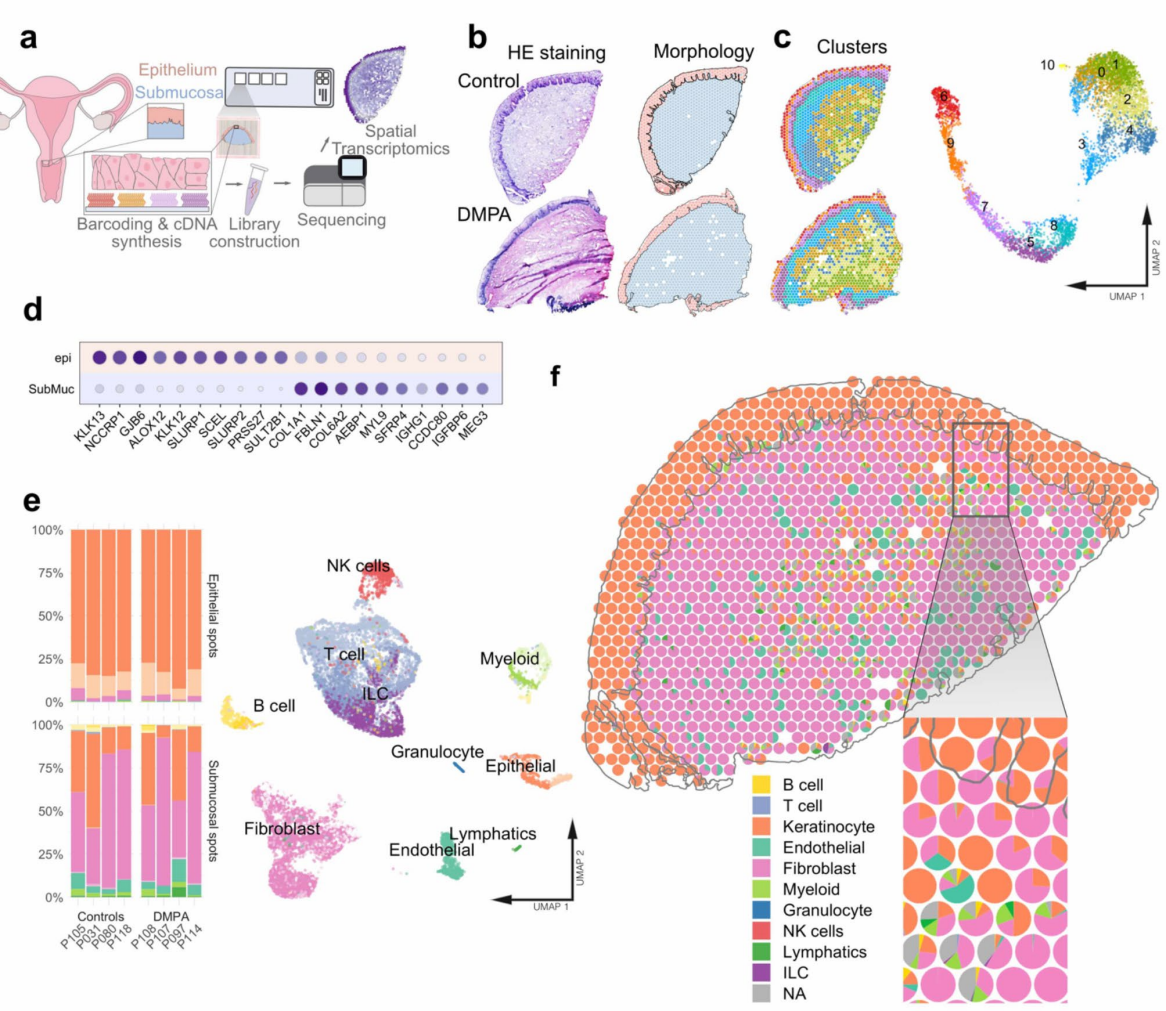
Overview of the study and transcriptional profiles associated with ectocervical morphological structures and cell populations. (**a**) Eight ectocervical biopsies (controls (*n* = 4) and DMPA (*n* = 4)) were cross-sectionally sliced (10 μm) for analysis by spatial transcriptomics (ST). The sections were placed on Visium slides with a spot diameter of 55 and 100 μm distance between spots. After staining with hematoxylin and eosin (HE), images were captioned for each of the sections. Thereafter, the tissue was permeabilized with the enzyme pepsin allowing RNA to be released from the tissue and captured onto probes attached to the ST slide. After library preparation, captured mRNA was sequenced on the Illumina NextSeq 500/550 platform. (**b**) HE staining and manual annotation of two morphological regions, epithelium (blue) and submucosal (orange) (Pat-ID P118 and P097) (**c**) Classification by the unsupervised clustering visualized on tissue (left) (Sample-ID P118 and P097) and using Uniform Mani­fold Approximation and Projection (UMAP) (right). A total of 11 clusters (4 epithelial (“epi”) and 7 submucosal (“SubMuc”)) are depicted. (**d**) Top marker genes across the two morphological structures. (**e**) Proportion of predicted cell types per sample in the epithelium (top) and submucosa (bottom). (**f**) Deconvolution results plotted on tissue (Sample-ID P118).

DMPA use significantly influences the transcriptional profile of the ectocervical mucosa^9,10^. To map and spatially resolve these molecular associations, all tissue samples underwent processing for spatial transcriptomics (Fig. 1a). The mRNA expression across the tissue was delineated within individual “spots” measuring 55 μm in diameter, estimated to represent 1–10 cells. The resulting dataset from the eight study participants comprises 6,598 individual spots, with a median of 2,424 total transcripts and 1,355 genes per spot (Supplementary Fig. 1). Both study groups exhibited a similar distribution of spots (median: DMPA: 2,933; Controls: 3,665), total transcripts/counts (median: DMPA: 2,585; Controls: 2,279), and genes/features (median: DMPA: 1,422; Controls: 1,276).

Subsequently, we annotated the morphology to categorize the spots into epithelial and submucosal spots by manually outlining the shape of the mucosa (Fig. 1b, Supplementary Fig. 1). The spots were then grouped based on gene expression using unsupervised clustering^25^, revealing four epithelial (cluster 5–7, and 9) and seven submucosal (cluster 0–4, 8, and 10) clusters (Fig. 1c, Supplementary Fig. 1). Differential gene expression analysis between epithelial and submucosal spots across the eight samples resulted in a total of 7,239 upregulated and 1,005 downregulated genes (Supplementary Table 1). Notably, the epithelial spots demonstrated significant enrichment of keratinocyte markers and associated processes, while submucosal spots exhibited an enrichment of fibroblast activity^21,22^ (Fig. 1d).

### Spatial mapping of cell types across the ectocervical mucosal layers

**Fig. 2:**
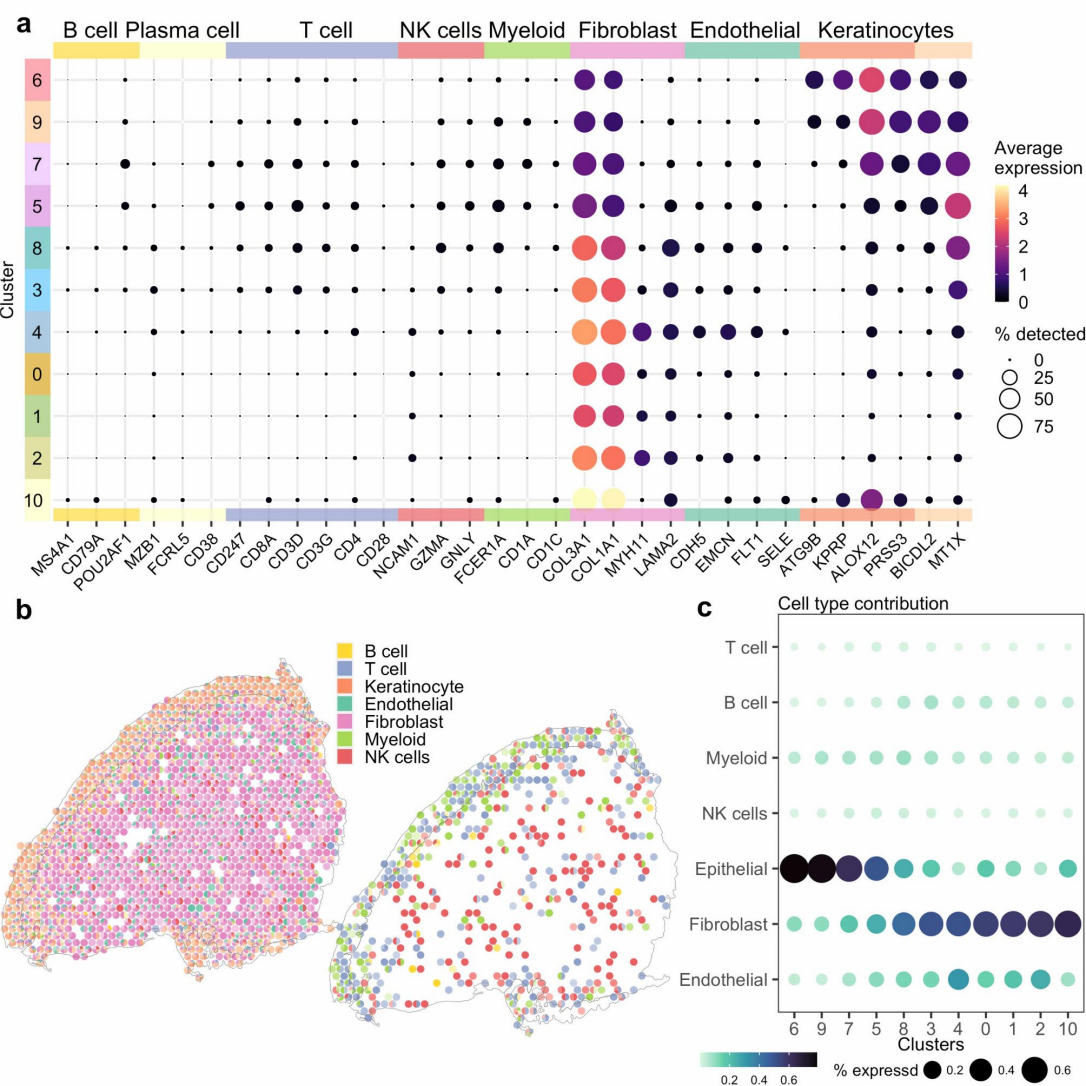
Cell populations in the ectocervix (**a**) Average expression of selected cell marker genes visualized for each of the 11 clusters (cluster 0–10). (**b**) Relative proportions of marker genes including immune, epithelial, fibroblast and endothelial markers (left) and immune markers only (right) (Sample-Id P097). (**c**) Estimated cell type contribution per cluster.

To assess the cell composition within each spot, proportional estimates of cell types were calculated by deconvolution using a single-cell dataset as reference^26^. All epithelial spots exhibited keratinocyte marker expression, identifying a minimum of 80% as keratinocytes. The submucosal spots displayed greater heterogeneity, characterized by an average distribution of spots identified as 61% fibroblasts, 24% keratinocytes, 9% immune cells, and 6% endothelial cells (Fig. 1e,f, Supplementary Fig. 1, Supplementary Table 2). Certain keratins were found to be expressed in the submucosa. These, along with other markers associated with keratinocytes, likely expressed by fibroblasts, may explain the prediction of keratinocyte presence in specific regions of the submucosa^27,28^. Expression of cell marker genes, primarily selected from Ou et al.^22^ was assessed within each cluster to corroborate the deconvolution results (Fig. 2a). Markers for immune cells, including T cells (CD247, CD8A, CD3D, CD3G, CD4, CD28) and NK cells (NCAM1, GZMA, GNLY), showed the highest expression at the interface of the epithelial and submucosal layers, aligning with the deconvolution findings (Fig. 2b,c).

### Spatial ordering of the ectocervical mucosal layers reveals functional stratification across all samples

**Fig. 3:**
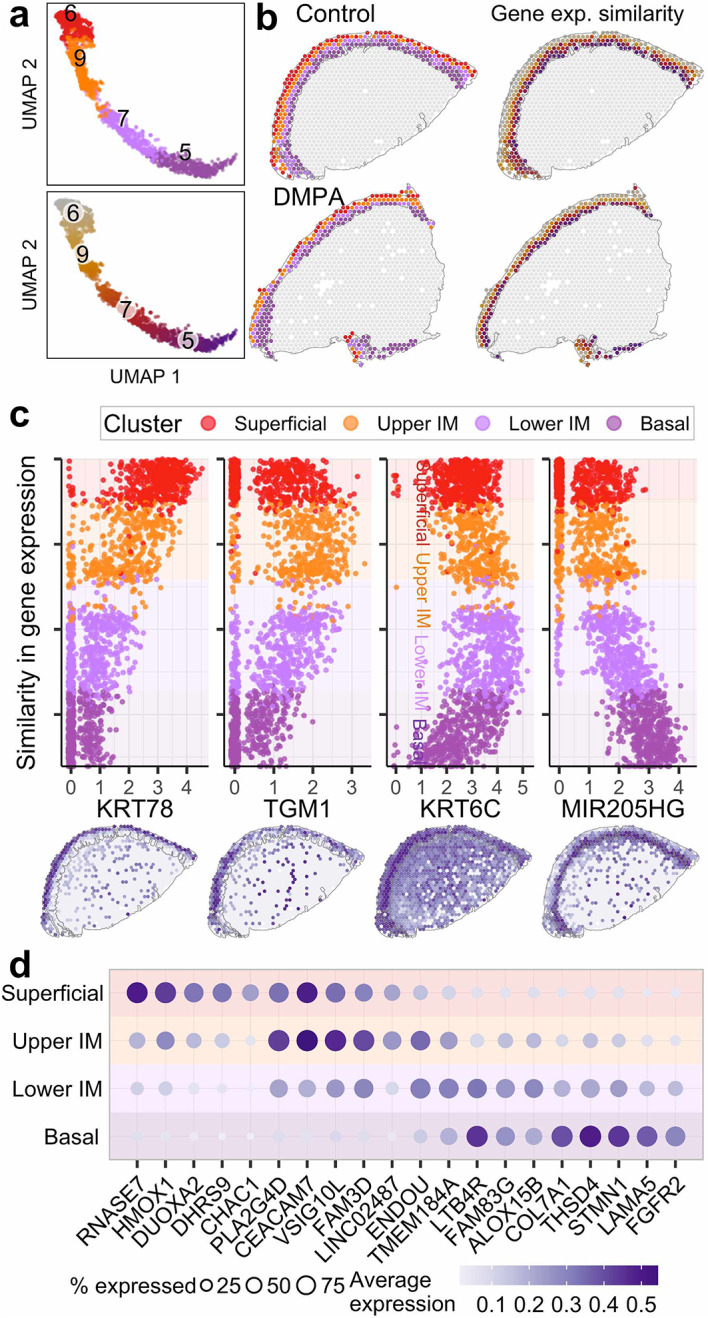
Gene expression patterns across the ectocervical epithelium (**a**) Uniform mani­fold approximation and projection (UMAP) of epithelial spots, colored by clustering (top) and the similarity in gene expression/gene expression trajectory (bottom). (**b**) Clustering (left) and similarity in gene expression (right) plotted on the tissue of control sample P118 and DMPA sample P097. (**c**) On the y-axis, spots are ordered by similarity in gene expression, resulting in a pseudo measurement (0–20) of distance across the epithelium. The x-axis displays the gene expression for selected marker genes representing the superficial, upper intermediate (IM), lower IM and basal layers of the epithelium, respectively. The same genes highlighted on tissue of control sample P118. (**d**) Top five marker genes for the four epithelial clusters.

By employing trajectory analysis, the spatial ordering of the epithelial layers was delineated in a continuum, progressing from the outer to the innermost layers (Fig. 3a,b). Trajectory analysis validated the close correlation of gene expression patterns and spatial distances relative to the basal membrane, mirroring the function of each specific layer. Marker genes corresponding to the respective layer demonstrated distinct transcriptional patterns (KRT78, TGM1, KRT6C, MIR205HG) (Fig. 3c). The top five marker genes within each epithelial layer exhibited distinct functions (Fig. 3d, Supplementary Table 3). In the superficial layer, genes were involved in reactive oxygen species (ROS)-associated processes and innate antimicrobial responses (RNASE7, HMOX1), while upper-intermediate layer genes reflected terminal differentiation and immune responses (PLAG2GAD, FAM3D). The lower-intermediate layer was characterized by genes associated with cell differentiation (ENDOU, ALOX15B) and the basal layer with basal membrane function and cell division (LAMA5, FGFR2).

**Fig. 4:**
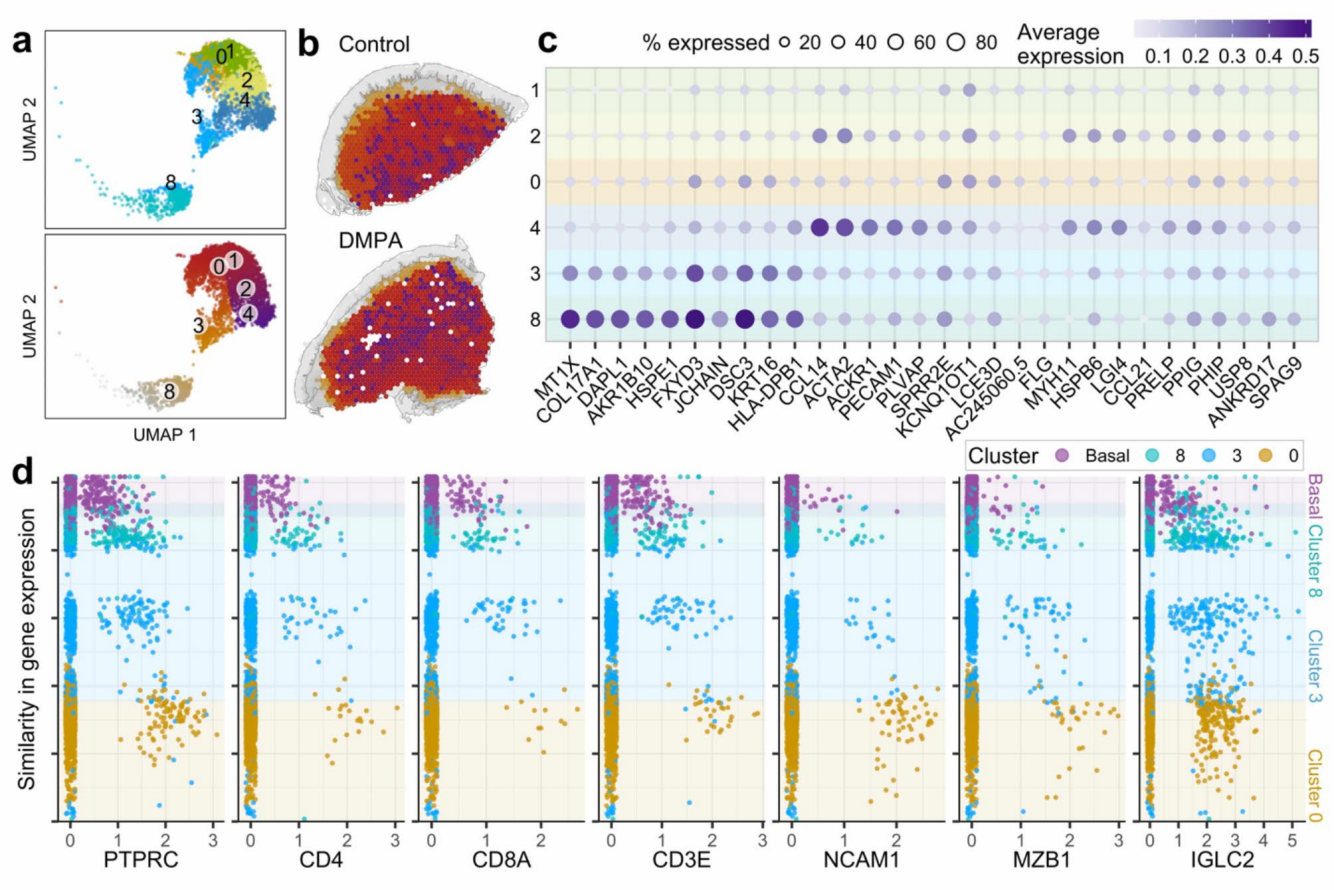
Gene expression patterns across the ectocervical submucosa (**a**) Uniform manifold approximation and projection of submucosal spots, colored by clustering (top) and the similarity in gene expression/gene expression trajectory (bottom). (**b**) Similarity in gene expression plotted on the tissue of control sample P118 and DMPA sample P097. (**c**) Top five marker genes for the six submucosal clusters (cluster 10 was excluded from analysis). (**d**) On the y-axis, spots are ordered by similarity in gene expression, resulting in a pseudo measurement (0–25) of the distance from the basal layer into the submucosa. The x-axis displays the gene expression for selected marker genes representing the basal epithelial layer (purple), cluster 8 (turquoise), cluster 3 (light blue), cluster 0 (mustard).

Spatial ordering of the submucosal layers demonstrated a less organized structure and greater inter-individual differences compared with the epithelium across all samples (Fig. 4a,b, Supplementary Fig. 1). Cluster 10, which was almost exclusively expressed in a single sample (P107), was not included in further analysis. Marker gene analysis for the submucosal clusters showed more overlap in function between clusters than for the epithelial layers. While clusters 1 and 0 had no clear marker genes (Fig. 4c, Supplementary Table 3), they expressed fibroblast markers, interpreted as less cell-dense areas of fibroblasts. The marker genes of cluster 4 were associated with endothelial and smooth muscle function, which aligns with the more dispersed placement of this cluster.

The submucosal region is typically populated by immune cells^29^. We focused our attention on clusters 8, 3, and 0, which exhibited spatial continuity in proximity to the basal membrane. The general leukocyte marker PTPRC/CD45 confirms that the highest concentration of immune cells was found in cluster 8 with many cells also present in the basal epithelium (Fig. 4d). CD4, CD8A, CD3E, and NCAM1 expression provided an approximate distribution of T cells and natural killer (NK) cells. The NK cells were more distally distributed from the basal membrane and were barely present in the basal epithelium, while T cells were abundant in cluster 8 and the basal epithelium. Interestingly, we noted a difference between the expression of the innate-like B-cell marker MZB1, and IGLC2, with the latter having stronger expression in clusters 8 and 3 as compared with the more random distribution of MZB1.

Collectively, the spatial analysis of ectocervical mucosal layers demonstrates a clear functional stratification, reflected by distinct marker gene expression patterns corresponding to specific epithelial layers, while submucosal layers exhibit greater variability and overlapping functions between clusters, underscoring the complexity of cellular composition within this region.

### Differential gene expression associated with DMPA use exhibits a distinct spatial distribution across epithelial layers

After thoroughly examining transcriptional patterns in all study samples, our focus shifted to identifying DMPA-associated effects by comparing the two study groups. Overall, the deconvolution analysis did not reveal any significant differences in proportion of various cell populations (DMPA vs. control: *p* > 0.05) (Supplementary Table 2).

**Fig. 5:**
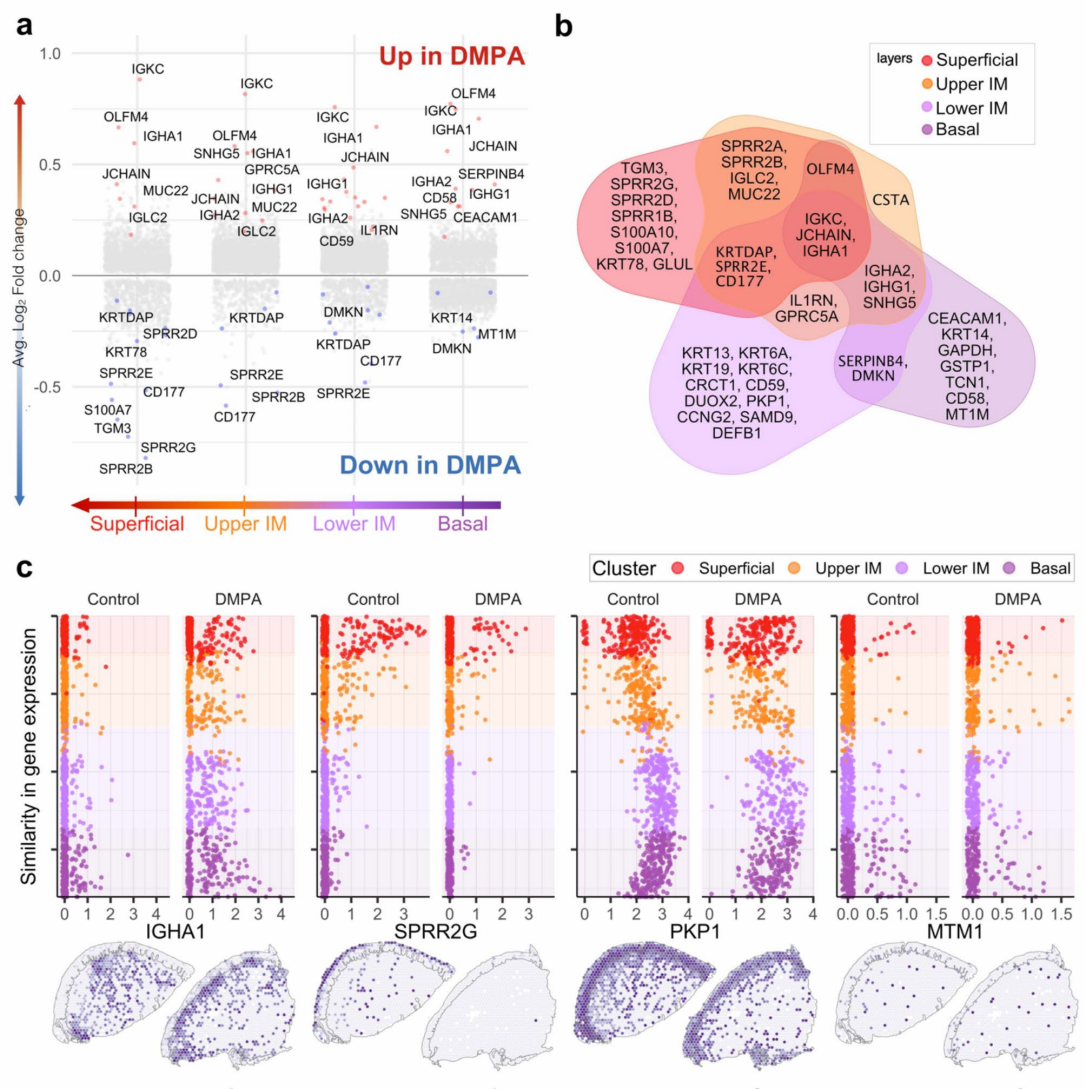
Differentially expressed genes associated with DMPA use across the ectocervical epithelium. (**a**) Volcano plots of each epithelial cluster on the x-axis and log fold change on the y-axis. (**b**) Venn diagram of the significant DEGs in the epithelium. (**c**) The gene expression (x-axis) of selected genes, plotted against the similarity in gene expression/gene trajectory (y-axis), including all samples from the respective study group (top). Gene expression values plotted on tissue slides of control sample P118 and DMPA sample P097 (bottom).

Examining the DMPA-associated effects across all four epithelial clusters, we identified 45 upregulated and 31 downregulated differentially expressed genes (DEGs) with a false discovery rate (FDR) < 0.05 (Fig. 5a,b, Supplementary Table 4). Upregulated genes were mainly associated with humoral and innate immune responses, skin development, and cell proliferation. While immunoglobulin genes (IGHA1, IGKC, and JCHAIN) were universally upregulated across all four layers, innate immune-related genes were predominately upregulated in the lower-intermediate and basal layers (Supplementary Table 4).

The downregulated genes exhibited a more nuanced pattern, with most directly or indirectly involved in maintaining epithelial barrier integrity. In the superficial layer, the downregulation of several cornified envelope crosslinking genes was predominant (SPRR1B, SPRR2A/B/D/E/G). In the upper-intermediate layer, gene expression changes showed similarities to the superficial layer, although less pronounced. In the lower-intermediate layer, downregulated genes, such as keratins and PKP1, are involved in stabilizing epithelial junctions. In the basal layer, downregulated genes, including MTM1 (transcriptionally regulated by glucocorticoids), displayed diverse functions (Fig. 5c).

Expanding on the gene expression results, Gene Set Enrichment Analysis (GSEA) with the gene ontology (GO) database (Supplementary Table 5) revealed six prominent GO-groups of terms: Immunity (GO1-2, and GO6), intracellular transport (GO3), transcriptional regulation (GO4-5), aerobic respiration (GO7), extracellular matrix organization (GO8), and keratinization (GO9) (Supplementary Fig. 2). Adaptive immune-related terms and keratinization showed consistent representation across all layers, while intracellular transport and transcriptional regulation were predominantly found in the upper intermediate layer. Extracellular matrix organization terms were notable in the lower intermediate layer, and aerobic respiration terms were primarily concentrated in the basal layer.

Collectively, DMPA associated with upregulation of immunoglobulin genes across the epithelium, whereas the upregulation of immune markers, skin development and cell proliferation were more spatially restricted. The DMPA-associated downregulated genes were also spatially restricted and represented foremost disrupted epithelial barrier integrity.

### Differential gene expression associated with DMPA use reveals altered immune functions and tissue remodeling processes in the submucosa

**Fig. 6:**
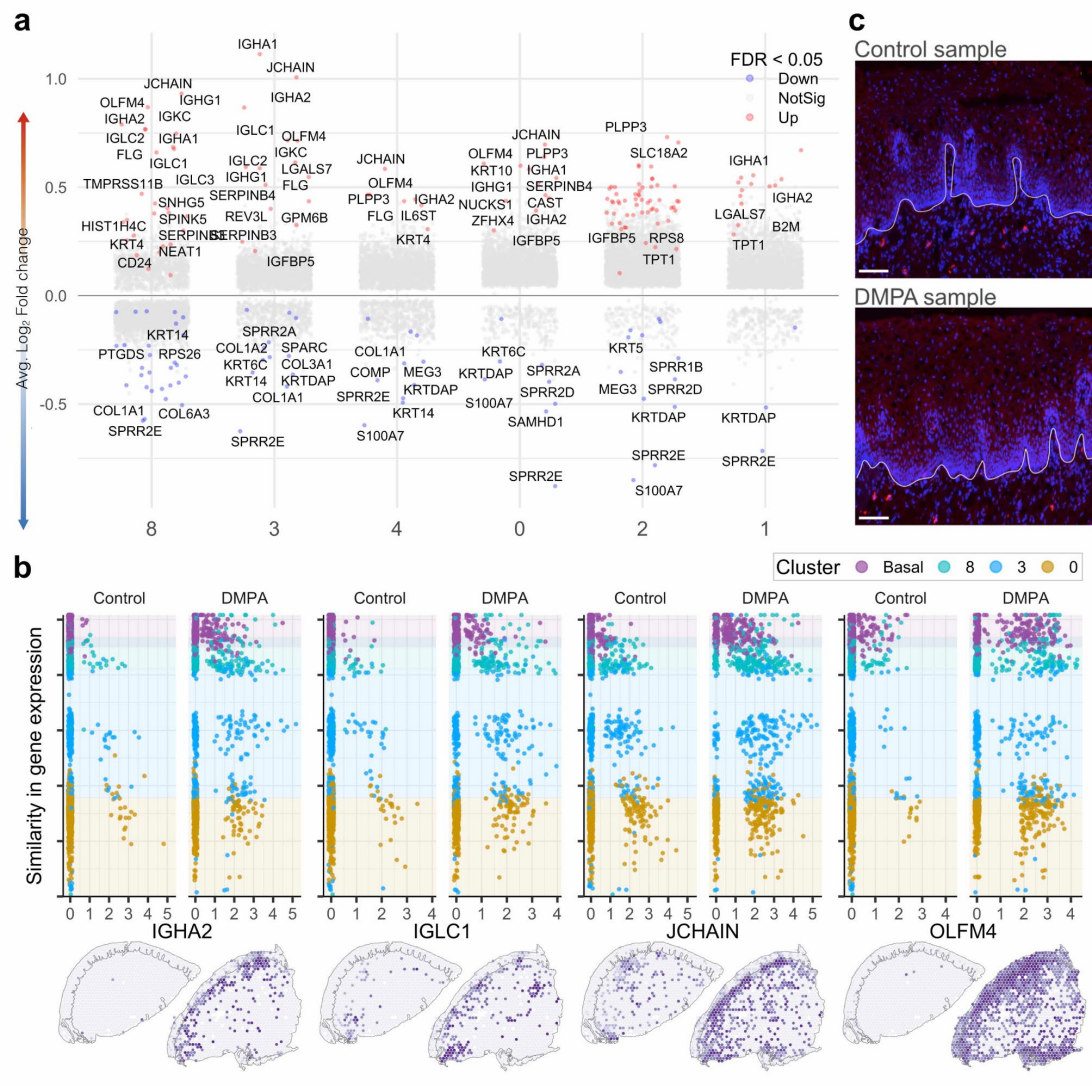
Differentially expressed genes associated with DMPA use across the ectocervical submucosa. (**a**) Volcano plots of each epithelial cluster on the x-axis and log fold change on the y-axis. (**b**) The gene expression (x-axis) of selected genes, plotted against the similarity in gene expression/gene trajectory (y-axis), including all samples from the respective study group (top). Gene expression values plotted on tissue slides of control sample P118 and DMPA sample P097 (bottom). (**c**) Immunofluorescent staining of CD20 + B cells (red) in a representative control sample P118 (top) and DMPA sample P097 (bottom). The scale bar is 1000 μm. The apical layer of the epithelial basal membrane is marked in white. Contrast has been enhanced for visualization purposes.

Analyzing submucosal clusters, the investigation identified 130 upregulated and 73 downregulated genes when comparing the DMPA and control groups (FDR < 0.05) (Fig. 6a, Supplementary Table 4). Several immunoglobulin genes were upregulated in clusters 3 and 8, signifying an impact on immune processes. Another set of upregulated DEGs, including OLFM4, represented innate immune-related functions (Fig. 6b). Although unique cluster-specific upregulated DEGs were less prominent, most DMPA-associated effects were closer to the epithelial basal membrane. To ascertain whether the heightened expression of immunoglobulin genes in the DMPA study group correlated with the presence of B cells in the ectocervical tissue samples, we stained corresponding tissue sections for CD20. CD20^+^ B cells were sparsely distributed in the epithelium, while more abundant in the submucosal layers, predominantly near the epithelial basal layer. This cellular distribution pattern substantiates the spatially confined expression of immunoglobulin genes (Fig. 6c).

Focusing on downregulated DEGs in the submucosa, we observed a prevalence of components related to the extracellular matrix, indicating potential alterations in tissue structural support (Supplementary Table 4). Downregulated genes crucial for tissue structure included members of the small proline rich (SPRR) and keratin families. Cluster 8, which exhibited the broadest range of downregulated genes, along with clusters 3 and 4, showed a reduction in COL1A1/3A1/1A2. This downregulation may reflect their higher cell density and a large proportion of fibroblasts compared to other submucosal clusters.

GSEA within the submucosal context revealed significant differences in numerous GO terms between the DMPA and control groups (Supplementary Table 5). Examining clusters 8, 3, 4, and 0, cluster 0 emerges with the most terms across nearly all GO clusters (Supplementary Fig. 3) Nevertheless, all clusters contain terms related to innate and adaptive immune response (GO2-4). Unlike Cluster 8 and 4, which have more unique functional groups of terms, Cluster 3 is primarily associated with immune-related terms, including responses to bacteria (GO2), and viral lifecycle (GO11). Additionally, a significant proportion of submucosal GO-terms are linked to various stages of protein expression dynamics (translation, modification, localization), suggesting an overall increase in metabolic processes (GO5-10). Notably, Cluster 8 is primarily associated with GO9, while Cluster 4 contributes to GO6, 7, and 10. Terms such as “regulation of glucocorticoid biosynthetic process” (cluster 4) and “glucose-6-phosphate metabolic processes” (cluster 8) highlight distinct aspects of cellular metabolism, potentially associated with the off-target effects of DMPA on glucocorticoid receptors^17^.

In summary, DMPA-associated upregulation of innate immune marker and immunoglobulin genes, and corresponding staining for CD20 + B-cells, predominated near the epithelial basal layer. Downregulation of genes involved in structural tissue support suggested functional impairment of the submucosa, a finding reflecting the higher spatial resolution provided here as compared with our previous bulk transcriptomics study^10^.

### Analysis of differential gene expression in high vs. low plasma estradiol levels in the DMPA group

**Fig. 7:**
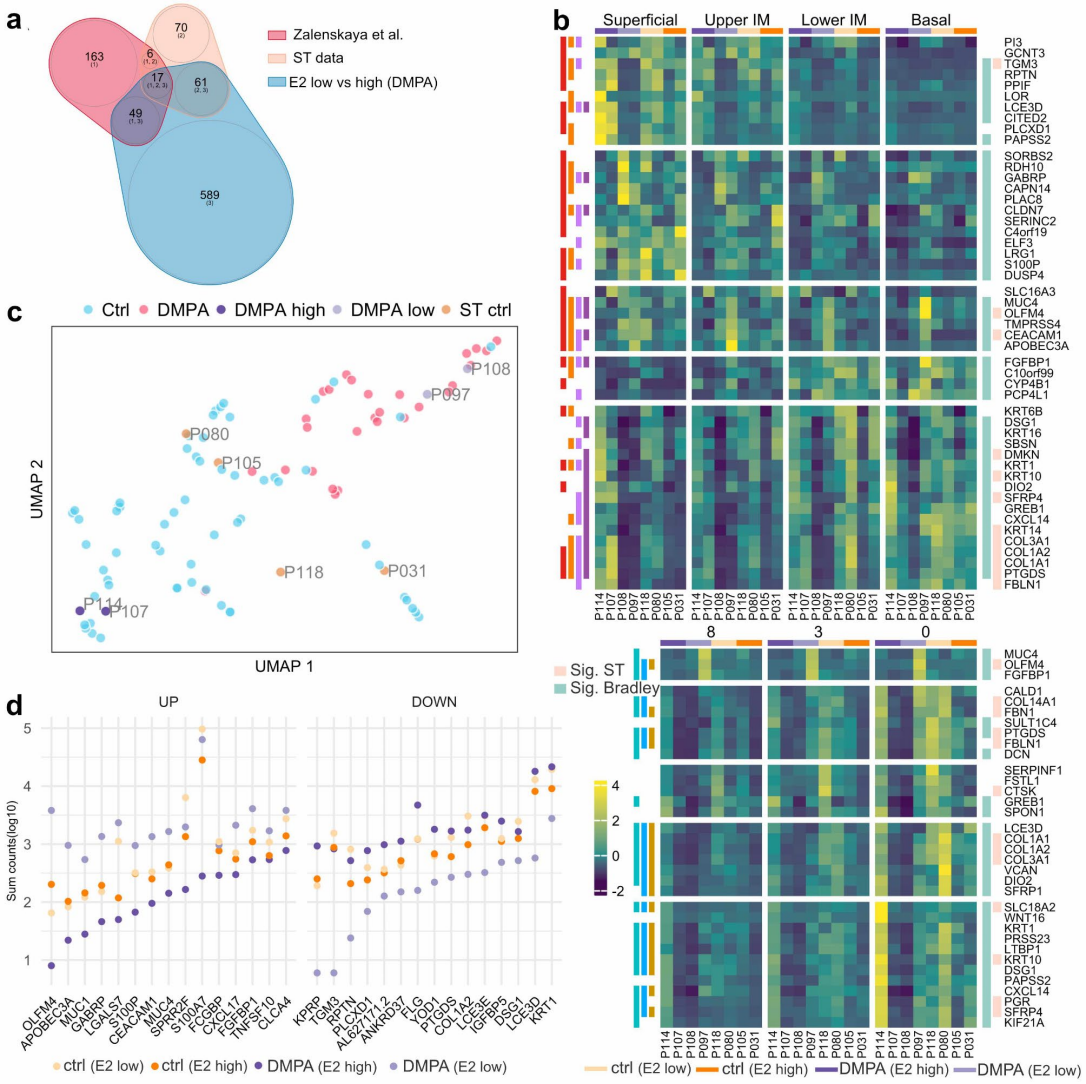
Estradiol (E2) low vs. high DEGs analysis and how they relate to bulk transcriptomics data. (**a**) Venn diagram highlighting the shared DEGs between Zalenskaya et al.^9^, and our spatial transcriptomics (ST) data (DMPA vs. controls), and the E2 low vs. E2 high (DMPA samples). (**b**) Heatmap of ST data showing the average gene expression across epithelial/submucosal clusters for each sample. The selected genes overlap between Zalenskaya et al.^9^ and E2 low vs. E2 high significant genes. The vertical lines on the left side of the plot indicate in which epithelial/submucosal layer the DEG reached significance. The vertical lines on the right indicate significance in Bradley et al.^10^ (green) and DMPA vs. controls (beige). (**c**) UMAP of bulk data illustrating how the current study’s samples are positioned within the broader cohort, showing control samples (ST ctrl) in yellow, DMPA (E2 low) in light purple, and DMPA (E2 high) in dark purple. (**d**) Log-normalized counts of the ST data, summarized across epithelial clusters, showing the top 15 upregulated and downregulated genes in the E2 low vs. E2 high comparisons.

Given the well-established link between DMPA and hypoestrogenism, we explored whether DMPA may exert its influence through additional mechanisms. We compared transcriptional patterns between samples with low (n = 2; indicative of high DMPA concentration) and high (n = 2; within the range of the control group; 22–405 pg/mL^10^) plasma estradiol levels, revealing a significant difference in gene expression (n = 1,409 DEGs) (Supplementary Table 4). Among these DEGs, “estrogen-independent” genes (n = 78) were defined as the overlap of estradiol low vs high (within the DMPA group) and DEGs between the DMPA vs control groups (all eight samples). The remaining genes (n = 638) were classified as “estrogen-dependent” (Fig. 7a). Our use of the term ‘estrogen-independent’ should be understood as specific to the context of this analysis and not as a definitive characterization of molecular function. Significant epithelial estrogen-independent genes (n = 17) included some immunoglobulin genes (IGKC, JCHAIN, IGHG1) and several small proline-rich genes (SPRR2A, SPRR2B, SPRR2D, SPRR2G). Significant submucosal estrogen-independent genes (n = 66) represented extracellular matrix functions (TSKU, LUM, COL5A1, THBS2, PLPP3, FGFR1, COL6A3, COL5A1), structural proteins (VIM, CAST, GPM6B, SNHG6, KRT4/5/13), and proliferation (MEG3, PTN, CAV1, PDS5B, SSBP3, CLIC4, LPAR1, THAP12).

Estrogen-dependent genes (Fig. 7b) comprised several DEGs indicative of an inflammatory environment as well as structural changes observed across the epithelial layers. This includes genes crucial for epithelial integrity, such as KRT1, LOR, RPTN, DSG1, and LCE3D, antimicrobial peptides (DEFB/1/4A, APOBEC3A), retinoic acid metabolism (RDH10, DHRS3 ALDH1A3), and ROS-related genes (DUOX1/2, HSPA4/5/8, HSPB1, PRDX5). Among the submucosal estrogen-dependent DEGs were genes associated with immune response and proliferation, with a majority significant for cluster 8 (closest to the basal membrane). Notably, these DEGs differed from the DMPA versus control comparison by lacking SPRR family genes. Additionally, the expression patterns in submucosal DEGs did not display a consistent trend between low and high estradiol samples among the selection of genes shown in (Fig. 7b).

In essence, the analysis highlighted a significant influence of estradiol levels on gene expression patterns in the ectocervical mucosa, revealing distinct profiles of estrogen-independent and estrogen-dependent genes.

### Consistency of findings: a comparison between the present spatial transcriptomics data and a previous bulk transcriptomics data set

We proceeded to compare the spatial transcriptomics data set (*n* = 8) with the bulk transcriptomics data set from the larger cohort (DMPA, *n* = 32; Control: *n* = 64), from which our samples were selected^10^ (GEO accession ID GSE183513). Average gene expression values from each sample plotted against the corresponding bulk data, displayed robust correlations across all samples (correlation coefficient > 0.7) (Supplementary Fig. 4). This overall similarity and consistency of our spatial samples with the bulk samples reinforce the reliability of the findings when contextualized within the broader dataset. By leveraging information from the bulk data set we get the comparative relationships among individual samples within their respective study groups (Fig. 7c). Notably, the two DMPA samples exhibiting low estradiol levels aligned seamlessly within the overall cluster of DMPA samples, whereas the two DMPA samples exhibiting high estradiol levels clustered alongside the control group. Furthermore, nearly all the top 15 up- and downregulated genes in the epithelial E2 comparisons exhibited opposite fold change in DMPA samples with low versus high estradiol levels relative to the control samples (Fig. 7d). This suggests a robust estrogen-dependent component, potentially explaining why certain DEGs observed in the larger cohort do not attain significance in the present study.

To further validate our findings, we compared our present spatial transcriptomics data with bulk transcriptomics data from the pivotal and independent Zalenskaya et al. study^9^, where 235 DEGs were identified in paired samples at baseline vs. after 6 weeks of DMPA treatment. The estrogen-dependent genes defined in our present study showed a notable overlap with the DEGs identified in the Zalenskaya transcriptomics study (Fig. 7a). For instance, genes such as KRT1, LOR, RPTN, DSG1, and LCE3D, which exhibited high log fold changes (logFC) in the Zalenskaya study, did not show significant differential expression in our DMPA vs. control comparison (Fig. 7b). However, in our DMPA estradiol low vs. estradiol high comparison, these and additional “estrogen-dependent” DEGs were consistent with findings in the bulk transcriptomics studies. This suggests strong estrogenic regulation dynamics within the DMPA study group. Other genes displaying similar dynamics include PGR (Fig. 7b). PGR codes for the progesterone receptor that binds the progestin component of DMPA, and therefore likely contributes to the sex hormone ligand/receptor feedback loops in the genital mucosa.

The overlap of estrogen-dependent and estrogen-independent DEGs observed in our study, alongside two additional studies^9,10^, underscores the validity of our sample selection despite the limited sample size.

## Discussion

In this spatial transcriptomics analysis of the ectocervical mucosa, we uncovered distinct molecular signatures within different layers of the epithelium and submucosa of a Kenyan sex worker cohort, shedding light on the functional implications of DMPA use. The most pronounced DMPA-associated finding was the overexpression of immunoglobulin genes, an observation not identified in the bulk analysis of the same cohort^10^. We also identified spatially restricted DMPA-associated activation of innate immune responses, epithelial development and cell proliferation as well as dysregulated epithelial barrier integrity. The hypoestrogenic state induced by DMPA emerges as a key factor influencing some of these molecular responses.

Our study stands out by examining gene expression in both morphological compartments of the ectocervical mucosa, distinguishing it from previous single-cell RNA sequencing or spatial transcriptomics studies which have focused on other aspects, particularly in the context of human papilloma virus-associated cancer^21,22,26^. As assessed across all samples in the present study, the spatial organization of the ectocervical epithelium revealed a nuanced functional stratification, similar to that of human skin^30^. Significantly, the superficial layer emerged as a hub for processes associated with ROS and innate antimicrobial responses, crucial elements in the mucosal defense against pathogens. The upper-intermediate layer primarily featured terminal differentiation and immune responses, indicating the potential interplay between cellular maturation and immunological defense mechanisms. The lower-intermediate layer, marked by active cell differentiation, and the basal layer, with its focus on basal membrane function and cell division, contributed to the dynamic cellular landscape. In contrast, the submucosa’s less structured spatial ordering and greater inter-individual differences underscored the complexity of this microenvironment. While T-cell genes were concentrated adjacent to the epithelial basal membrane, NK cell-related genes were more distally distributed within the submucosa.

Moving on to the DMPA-associated findings in the data set, the overexpression of immunoglobulin genes indicated a B-cell population in the ectocervical mucosa. The presence of B cells was further confirmed through immunostaining of CD20. The increased expression of antibody-related genes was evident across the epithelium of women using DMPA and more sporadically within clusters in the submucosa, with the highest expression near the basal membrane. Given the abundance of antibody-related gene expression, we hypothesize that these differences are likely due to the increased presence of plasma cells. Alternatively, the *MZB1* gene marker suggests that innate-like B cells could explain the observed differences. An increased presence of either of these cells could arise from DMPA-associated B cell dysregulation in response to chronic inflammation^10,31^, or tissue repair mechanisms as demonstrated in the gut^32^. Other genes upregulated in the epithelium in response to DMPA were associated with innate immune responses predominantly in the lower-intermediate and basal layers, as well as spatially restricted gene expression associated with epithelial development and cell proliferation. The high spatial resolution further unveiled DMPA-associated downregulated genes. In the superficial epithelial layer, there was a DMPA-associated downregulation of the TGM3 gene, along with several SPRR and KRT family genes. The upper-intermediate epithelial layer displayed a similar but less pronounced profile of downregulated genes. In the lower-intermediate region, downregulation primarily affected epithelial junction stabilizing functions, while the basal layer exhibited only a limited number of downregulated genes. Consistent with two other bulk transcriptomics studies^9,10^, we thus verified an overall DMPA-associated downregulation of genes representing epithelial barrier integrity. The overlap between the two bulk transcriptomics studies is noteworthy given the differences between them: non–sex workers vs. sex workers, American (mixed ethnicity) vs. Black population, and long-term vs. short-term DMPA use, as well as differing experimental techniques. By applying spatial transcriptomics technique, we could further demonstrate an impairment in structural support within the submucosa, in addition to the findings within the epithelium.

We propose that most DMPA-associated transcriptomic disparities resulted from the hypoestrogenic state that follows after DMPA injection, placing some individuals in a postmenopausal range of estradiol levels^33^. Over the 3-month interval between regular DMPA injection doses, DMPA concentrations gradually decrease, accompanied by a slow rise in estradiol levels. Our DMPA samples represented this spectrum, including both low and high estradiol levels, enabling a nuanced examination of estrogen-associated effects on the ectocervical transcriptional profile. The analyses revealed a significant influence of estradiol levels on gene expression patterns, mirroring trends observed in the DMPA users versus control comparison. As the active progestin component of DMPA binds to both the progesterone and glucocorticoid receptor^17^, DMPA may also impact the ectocervix through estrogen-independent mechanisms, potentially driven by off-target glucocorticoid effects, although these have not been fully established in humans. The identification of estrogen-independent genes in this study encompassed small proline-rich genes, collagens, and most immunoglobulin genes. Given the interconnected effects of glucocorticoids on the hypothalamic-pituitary-gonad axis, speculating about an estrogen-independent mechanism is challenging as estrogen can act as a counterforce against many of the glucocorticoid receptor effects. The altered immune responses could also be an indirect effect of the DMPA-associated breach of the epithelial barrier integrity, allowing penetration of microbes and antigens.

The DMPA associated compromise of the mucosal integrity raises concerns about increased susceptibility to sexually transmitted infections. The altered distribution of CD4 Th17 cells, identified as a mucosal target cell population for HIV infection, was evident in women using DMPA within a prospective clinical trial of hormonal contraceptive compounds^34–36^, suggesting an elevated risk of HIV infection. Therefore, it is important to identify potential off-target molecular effects that DMPA may exert on the cervicovaginal mucosa, especially among women at high risk of sexually transmitted infections. This concern is underscored by the prevalent use of DMPA in HIV-endemic geographical areas^1^.

The present study exhibits several strengths. Firstly, our study population represented participants at a heightened risk of sexually transmitted infections, which enhances the relevance of our findings. Secondly, the study participants lacked bacterial vaginosis which has a significant influence on the ectocervical transcriptome. The study groups had comparable cervicovaginal microbiome compositions dominated by *Lactobacillus iners* which is representative of this study population^24^. Thirdly, as one of the pioneering studies leveraging spatially resolved transcriptomics data to identify marker genes across the human ectocervix, our findings revealed a striking resemblance to observations in skin studies^37^. Collectively, spatial transcriptomics adds value by enabling genome-wide, unbiased spatial analyses, uncovering insights beyond the scope of traditional methods like in situ hybridization or immunohistochemistry, and providing a standardized platform that simplifies experimental workflows. Despite these strengths, the study has certain limitations. The data are derived from an observational cohort, and therefore we cannot imply a causative role of DMPA in the differences observed in gene expression between the study groups. From a functional perspective, the identified mRNA levels may not always correlate perfectly with protein expression. The exclusive expression patterns observed in protein expression^38,39^ may be represented here as more gradual gradients at the gene expression level. In addition, the selection of samples from the larger cohort was based on the quality of the RNA and the mucosal morphology. This selection could theoretically introduce bias as DMPA may affect the mucosal structure. Furthermore, the relatively modest sample size, though consistent with similar studies employing spatial transcriptomics^40^, warrants acknowledgment. The identification of elevated estradiol levels in two DMPA samples serves as a double-edged sword: it allowed us to identify estradiol-associated genes but also introduced a dampening effect on the overall DMPA signal. Consequently, disentangling genuine biological signals from potential sample batch effects becomes challenging. Notably, genes such as FLG, KRT10, COMP, and SPINK5— that exhibited susceptibility to the influence of a singular sample also demonstrated significance in the broader cohort, highlighting their biological relevance. Interestingly, these genes had divergent fold changes in the spatial data. This phenomenon might be attributed either to heightened per-cluster resolution or to the implications of a limited sample selection. In this case, a larger sample size is required to clarify these trends. Finally, CD20 was selected to visualize the B cell population using immunostaining. However, this marker is downregulated on plasmablasts and plasma cells as these are likely contributing to the DMPA-associated increase of antibody-related genes and thus we could not perform any comparative analysis of the ectocervical plasma cell population.

The study presents novel insights into the human ectocervical mucosal structure and function, unveiling significant alterations in the spatially defined transcriptomic profile associated with DMPA use. These changes encompassed the altered expression of immunoglobulin genes which had not been identified in similar studies based on bulk transcriptomics^9,10^. Furthermore, modulation of genes related to mucosal barrier integrity was stratified and differentiated across the epithelium, and also observed in specific layers of the submucosa. Differential expression of genes linked to innate immune responses and metabolism was concentrated to the area adjacent to the basal membrane. The implications of these findings extend to mucosal health and susceptibility to infections, particularly HIV, shedding light on the molecular mechanisms underlying contraceptive effects. This study also complements previous spatial transcriptomics research on HPV-affected cervical tissue^21,22^, offering a comprehensive understanding of molecular dynamics in cervical health. Additionally, exploring the clinical implications of these molecular changes and their relevance to women’s health remains a crucial avenue for future investigation.

## Methods

### Study participants, clinical samples and data sources

Study participants were recruited as part of a larger longitudinal study within a cohort of Kenyan sex workers^10,41^. Inclusion criteria for the larger study included individuals aged 18–50 years who were not pregnant or breastfeeding, not menopausal, and had no history of hysterectomy. Inclusion criteria for the DMPA study group included documented DMPA use for at least 6 months. We aimed to sample 4–8 weeks after the last DMPA injection. Two ectocervical tissue biopsies, each measuring up to 3 mm^3^, were obtained from each participant by a senior gynecologist. Collected samples were immediately snap-frozen and stored at -80 °C in liquid nitrogen for subsequent analysis. The women agreed to abstain from sexual activity for a two-week period following tissue sampling for safety reasons. Compliance with this requirement was facilitated through a combination of text messages providing instructions, on-site detection of prostate-specific antigen (PSA), and monetary compensation for income loss^41^. A clinical examination was conducted for all participants 3–5 days post-biopsy to assess the healing process and confirm adherence to the instructions.

This study adhered to the principles outlined in the Helsinki Declaration and received approval from the ethical review boards at the University of Manitoba, the University of Nairobi’s Kenyatta National Hospital, and the regional Ethical Review Board in Stockholm. Written informed consent was obtained from all participants.

Bulk RNA-sequencing (RNA-seq) data from the ectocervical tissue biopsies representing the larger study cohort were previously published^10,24^, and are available in the Gene Expression Omnibus (GEO) public repository (accession ID GSE217237). Here, we utilized the gene expression counts for comparison between these counts and the present spatial transcriptomics data set. Estradiol levels in paired plasma samples were measured using an electrochemiluminescence immunoassay (Roche Diagnostics) at the accredited Karolinska University Laboratory, Stockholm, and were previously published^10^. The lower limit of detection for estradiol was 22 pg/mL.

### Sample processing and visium library preparation

Tissue sections, sliced to a thickness of 10 μm using a pre-cooled cryostat, were placed onto a Visium 10x Genomics slide, ensuring alignment within the 6.5-mm² oligo-barcoded capture areas. Standard hematoxylin and eosin (H&E) staining was applied after tissue fixation, followed by imaging on the slide. Library preparation for sequencing adhered to the manufacturer’s instructions (10x Genomics, Visium Spatial Transcriptomic). Sequencing was conducted on a NovaSeq6000 flowcell in ‘SP’ mode, with specification of 28 bases for Read 1, 10 bases for Index 1 and 2, and 90 bases for Read 2. The Bcl to FastQ conversion was carried out using bcl2fastq_v2.20.0.422 from the CASAVA software suite.

### Data preprocessing

Raw reads were processed utilizing the spaceranger command line tool (version 1.2.0, 10x Genomics) and mapped to the human reference genome (Homo sapiens, GRCh38-2020-A). Gene expression matrices and tissue images for each sample were loaded and transformed into a Seurat object using the load10x_spatial and Read10X_Image functions. The merged dataset underwent filtering, where spots with fewer than 100 genes and/or more than 15% mitochondrial genes (*n* = 14) were removed. Highly variable genes (HVGs) were identified in each sample using the FindVariableFeatures function (nfeatures = 2,000, method="vst”). HVGs were then filtered to retain only those genes that were present in at least two samples, excluding VDJ genes. The selected HVGs were subsequently normalized (NormalizeData, normalization.method="LogNormalize”) and scaled (ScaleData). This processed set of HVGs underwent principal component analysis (PCA) via the RunPCA function.

The resulting PCA embedding was used for integration across all samples using Harmony (reduction="pca”, assay.use="RNA”, dims.use = 1:50). For unsupervised clustering, the Harmony embedding was used with dimensions 1 to 30 and k.param = 15, applying a k-nearest neighbors (KNN) approach (via FindNeighbors and FindClusters functions from Seurat) at a resolution of 1.0 and using louvain algorithm. Data were embedded into a 2D Uniform Mani­fold Approximation and Projection (UMAP) using the first 50 harmony vectors as input to the RunUMAP function in Seurat. The epithelial, and submucosal spots were annotated by manually outlining the shape of the mucosa. The resulting polygon shapes were utilized to assign spots. Spots that were only partially covered by the polygons were manually assigned.

### Deconvolution of spatial transcriptomics data

Utilizing the SCDC package^42^, the spatially resolved transcriptomic dataset underwent deconvolution at two distinct levels of cell granularity. Marker gene expression used as a reference was extracted from a previously published single-cell RNA-seq dataset^26^. Initially, the spatial data were filtered for spots with < 200 features before being subsetted to ensure a more equal distribution of spots per cluster. Top marker genes were then selected for each cell type level.

### Differential gene expression and enrichment analysis

Differential gene expression analysis was conducted using FindAllMarkers (test.use = Wilcox) from Seurat to identify marker genes for each cluster, DEGs across conditions and the sub-study of estradiol low vs. high. This analysis was performed on a reduced version of the dataset, with a random sampling of a total of 25 and 50 spots from each epithelial and submucosal cluster, respectively, in each sample. For each cluster, Gene Set Enrichment Analysis (GSEA)^43^ was conducted. Genes were ordered based on their log fold change and subjected to enrichment analysis against the GO database^44,45^. Significant enrichment terms (p-adjusted < 0.05) were clustered (by Louvain algorithm) based on the Jaccard similarity coefficient for epithelial and submucosal terms respectively. The results were visualized as graphs by the packages igraph^46^ and tidygraph^47^. Edges were removed between terms with less than 30% gene overlap for the largest clusters.

### Spatial trajectory analysis

To elucidate the spatiotemporal dynamics of gene expression at the individual spot level, we utilized the slingshot package^48^. This tool facilitated the mapping of gene expression trajectory changes separately in both the epithelium and submucosa. Specifically, spots are ordered by similarity in gene expression, resulting in a pseudo measurement of distance across the mucosa.

### In situ immunofluorescence staining

In situ immunofluorescence staining was performed on 8 mm thick cryopreserved etocervical tissue sections to assess CD20 expression. The tissue sections were fixed in 2% formaldehyde (Sigma-Aldrich, Solna, Sweden). The primary CD20 antibody (Clone; 2H7, BD Biosciences, San Jose, CA, USA) was added to the tissue sections followed by an Alexa Fluor 647-conjugated donkey anti mouse secondary antibody (A-31571, Invitrogen, Thermo Fischer Scientific, Waltham, MA, USA). After each step, the tissue sections were washed with phosphate buffered saline containing 1% HEPES (HyClone, Nordic Biolabs, Täby, Sweden) and saponin (Sigma-Aldrich). Negative controls were incubated with only secondary flourophore conjugated antibody. All tissue sections were counterstained with 4’6-diamidino-2-phenylindole (DAPI; Invitrogen, Thermo Fischer Scientific). The tissue sections were scanned into digital images using a Pannoramic 250 Flash Slide Scanner with a 20x objective (3DHISTECH Ltd, Budapest, Hungary).

## Electronic supplementary material

Below is the link to the electronic supplementary material.

Supplementary Material 1

Supplementary Material 2

Supplementary Material 3

Supplementary Material 4

Supplementary Material 5

Supplementary Material 6

## Data Availability

The clinical characteristics of the study participants, as well as the raw counts of the spatial and bulk RNA sequencing data, are available at the gene expression omnibus (GEO) public repository (accession ID: GSE217237). The raw sequencing data from the study participants cannot be held in a public repository due to the sensitive nature of such personal data. Requests for data access can be made to the Karolinska Institutet Research Data Office (contact: rdo@ki.se), and access will be granted if the request meets the requirements of the data policy. All analysis of spatial and bulk RNA-seq is available at https://github.com/vildeka/Spatial_DMPA.

## References

[1] United Nation Department of Economic and Social Affairs. 25. Contraceptive use by method 2019: data booklet. Contraception Use by Method (2019).

[2] Butler, A. R. et al. Modelling the global competing risks of a potential interaction between injectable hormonal contraception and HIV risk. *AIDS* **27**, 105–113 (2013). 23014519 10.1097/QAD.0b013e32835a5a52 PMC4862571

[3] Plummer, F. A. et al. Cofactors in male-female sexual transmission of human immunodeficiency virus type 1. *J. Infect. Dis.* **163**, 233–239 (1991). 1988508 10.1093/infdis/163.2.233

[4] Morrison, C. S. et al. Hormonal contraception and the risk of HIV acquisition: an individual participant data meta-analysis. *PLoS Med.* **12**, e1001778 (2015). 25612136 10.1371/journal.pmed.1001778 PMC4303292

[5] Polis, C. B. et al. An updated systematic review of epidemiological evidence on hormonal contraceptive methods and HIV acquisition in women. *AIDS* **30**, 2665–2683 (2016). 27500670 10.1097/QAD.0000000000001228 PMC5106090

[6] Curtis, K. M. et al. Hormonal contraception and HIV acquisition among women: an updated systematic review. *BMJ Sex. Reprod. Health* **46**, 8–16 (2020). 31919239 10.1136/bmjsrh-2019-200509 PMC6978562

[7] Ahmed, K. et al. HIV incidence among women using intramuscular depot medroxyprogesterone acetate, a copper intrauterine device, or a levonorgestrel implant for contraception: a randomised, multicentre, open-label trial. *Lancet* **394**, 303–313 (2019). 31204114 10.1016/S0140-6736(19)31288-7 PMC6675739

[8] Hapgood, J. P. Is the injectable contraceptive depo-medroxyprogesterone acetate (DMPA-IM) associated with an increased risk for HIV acquisition? The jury is still out. *AIDS Res. Hum. Retroviruses* **36**, 357–366 (2020). 31797677 10.1089/aid.2019.0228 PMC7232639

[9] Zalenskaya, I. A. et al. Use of contraceptive depot medroxyprogesterone acetate is associated with impaired cervicovaginal mucosal integrity. *J. Clin. Invest.* **128**, 4622–4638 (2018). 30222141 10.1172/JCI120583 PMC6159996

[10] Bradley, F. et al. Multi-omics analysis of the cervical epithelial integrity of women using depot medroxyprogesterone acetate. *PLoS Pathog.* **18**, e1010494 (2022). 35533147 10.1371/journal.ppat.1010494 PMC9119532

[11] Dabee, S. et al. Update on the impact of depot medroxyprogesterone acetate on vaginal mucosal endpoints and relevance to sexually transmitted infections. *Curr. HIV/AIDS Rep.* **20**, 251–260 (2023). 37341916 10.1007/s11904-023-00662-0 PMC10403392

[12] Woods, M. W. et al. Transcriptional response of vaginal epithelial cells to medroxyprogesterone acetate treatment results in decreased barrier integrity. *J. Reprod. Immunol.* **143**, (2021). 10.1016/j.jri.2020.103253 33285485

[13] Liu, M. et al. Genital epithelial barrier function is conserved by intravaginal rings releasing etonogestrel and ethinyl estradiol. *Tissue Barriers* **12**, (2024). 10.1080/21688370.2023.2186672 PMC10832912 36899465

[14] Gupta, P. M. et al. Systems analysis reveals differential expression of endocervical genes in African women randomized to DMPA-IM, LNG implant or cu-IUD. *Clin. Immunol.* **255**, (2023). 10.1016/j.clim.2023.109750 PMC10570927 37660744

[15] Quispe Calla, N. E., Miguel, V., Aceves, R. D., Torres, K. M., Cherpes, T. L. & A. & Depot-medroxyprogesterone acetate reduces genital cell–cell adhesion molecule expression and increases genital herpes simplex virus type 2 infection susceptibility in a dose-dependent fashion. *Contraception* **100**, 397–401 (2019). 31302121 10.1016/j.contraception.2019.07.003 PMC6875619

[16] Carias, A. M. et al. Increases in endogenous or exogenous progestins promote virus-target cell interactions within the non-human primate female reproductive tract. *PLoS Pathog.* **12**, e1005885 (2016). 27658293 10.1371/journal.ppat.1005885 PMC5033389

[17] Hapgood, J. P., Kaushic, C. & Hel, Z. Hormonal Contraception and HIV-1 Acquisition: Biological mechanisms. *Endocr. Rev.* **39**, 36–78 (2018). 29309550 10.1210/er.2017-00103 PMC5807094

[18] Ståhl, P. L. et al. Visualization and analysis of gene expression in tissue sections by spatial transcriptomics. *Science* **353**, 78–82 (2016). 27365449 10.1126/science.aaf2403

[19] Ke, R. et al. In situ sequencing for RNA analysis in preserved tissue and cells. *Nat. Methods* **10**, 857–860 (2013). 23852452 10.1038/nmeth.2563

[20] Thul, P. J. et al. A subcellular map of the human proteome. *Science* **356**, 2017 (1979). 10.1126/science.aal3321 28495876

[21] Guo, C. et al. Spatiotemporally deciphering the mysterious mechanism of persistent HPV-induced malignant transition and immune remodelling from HPV‐infected normal cervix, precancer to cervical cancer: integrating single‐cell RNA‐sequencing and spatial transcriptome. *Clin. Transl Med.* **13**, (2023). 10.1002/ctm2.1219 PMC10040725 36967539

[22] Ou, Z. et al. Single-nucleus RNA sequencing and spatial transcriptomics reveal the immunological microenvironment of cervical squamous cell carcinoma. *Adv. Sci.* **9**, 2203040 (2022). 10.1002/advs.202203040 PMC9561780 35986392

[23] Edfeldt, G. et al. Regular use of depot medroxyprogesterone acetate causes thinning of the superficial lining and apical distribution of human immunodeficiency virus target cells in the human ectocervix. *J. Infect. Dis.* **225**, 1151–1161 (2022). 32780807 10.1093/infdis/jiaa514 PMC8974825

[24] Edfeldt, G. et al. Distinct cervical tissue-adherent and luminal microbiome communities correlate with mucosal host gene expression and protein levels in Kenyan sex workers. *Microbiome* **11**, 67 (2023). 37004130 10.1186/s40168-023-01502-4 PMC10064689

[25] Hao, Y. et al. Integrated analysis of multimodal single-cell data. *Cell* **184**, 3573–3587e29 (2021). 34062119 10.1016/j.cell.2021.04.048 PMC8238499

[26] Peng, T. et al. Distinct populations of antigen-specific tissue-resident CD8 + T cells in human cervix mucosa. *JCI Insight* **6**, (2021). 10.1172/jci.insight.149950 PMC8410090 34156975

[27] Knapp, A. C. & Franke, W. W. Spontaneous losses of control of cytokeratin gene expression in transformed, non-epithelial human cells occurring at different levels of regulation. *Cell* **59**, 67–79 (1989). 2477157 10.1016/0092-8674(89)90870-2

[28] Katagata, Y., Takeda, H., Ishizawa, T., Hozumi, Y. & Kondo, S. Occurrence and comparison of the expressed keratins in cultured human fibroblasts, endothelial cells and their sarcomas. *J. Dermatol. Sci.* **30**, 1–9 (2002). 12354414 10.1016/s0923-1811(02)00039-7

[29] Hirbod, T. et al. Abundant and superficial expression of C-type lectin receptors in ectocervix of women at risk of HIV infection. *J. Acquir. Immune Defic. Syndr.* **51**, 239–247 (2009). 10.1097/QAI.0b013e3181a74f89 19363450

[30] Candi, E., Schmidt, R. & Melino, G. The cornified envelope: a model of cell death in the skin. *Nat. Rev. Mol. Cell. Biol.* **6**, 328–340 (2005). 15803139 10.1038/nrm1619

[31] Di Pietro, A. et al. Targeting BMI-1 in B cells restores effective humoral immune responses and controls chronic viral infection. *Nature Immunology 2021 23:1* **23**, 86–98 (2021). 10.1038/s41590-021-01077-y 34845392

[32] Frede, A. et al. B cell expansion hinders the stroma-epithelium regenerative cross talk during mucosal healing. *Immunity* **55**, 2336–2351e12 (2022). 36462502 10.1016/j.immuni.2022.11.002

[33] Mishell, D. R. Jr. Pharmacokinetics of depot medroxyprogesterone acetate contraception. 381–390 (1996). 8725700

[34] Lajoie, J. et al. Increased cervical CD4 + CCR5 + T cells among Kenyan sex working women using depot medroxyprogesterone acetate. *AIDS Res. Hum. Retroviruses* **35**, 236–246 (2019). 30585733 10.1089/aid.2018.0188 PMC6434599

[35] Bunjun, R. et al. Initiating intramuscular depot medroxyprogesterone acetate increases frequencies of Th17-like human immunodeficiency virus target cells in the genital tract of women in South Africa: a randomized trial. *Clin. Infect. Dis.* **75**, 2000–2011 (2022). 35941737 10.1093/cid/ciac284 PMC9710690

[36] McKinnon, L. R. & Kaul, R. Quality and quantity: mucosal CD4 + T cells and HIV susceptibility. *Curr. Opin. HIV AIDS* **7**, 195–202 (2012). 22314505 10.1097/COH.0b013e3283504941

[37] Edqvist, P. H. D. et al. Expression of human skin-specific genes defined by transcriptomics and antibody-based profiling. *J. Histochem. Cytochem.* **63**, 129–141 (2015). 25411189 10.1369/0022155414562646 PMC4305515

[38] Uhlén, M. et al. Tissue-based map of the human proteome. *Science* 347, (2015). 10.1126/science.1260419 25613900

[39] The human proteome in cervix - The Human Protein Atlas. https://www.proteinatlas.org/humanproteome/tissue/cervix

[40] Bhalla, N. et al. Spatial transcriptomics of human placentas reveal distinct RNA patterns associated with morphology and preeclampsia. *Placenta* **139**, (2023). 10.1016/j.placenta.2023.07.004 37481829

[41] Lajoie, J. et al. Improving adherence to post-cervical biopsy sexual abstinence in Kenyan female sex workers. *Am. J. Reprod. Immunol.* **76**, 82–93 (2016). 27221472 10.1111/aji.12520 PMC5089664

[42] Dong, M. et al. SCDC: bulk gene expression deconvolution by multiple single-cell RNA sequencing references. *Brief. Bioinform.* **22**, 416–427 (2021). 31925417 10.1093/bib/bbz166 PMC7820884

[43] Subramanian, A. et al. Gene set enrichment analysis: a knowledge-based approach for interpreting genome-wide expression profiles. *Proc. Natl. Acad. Sci. USA* **102**, 15545–15550 (2005). 16199517 10.1073/pnas.0506580102 PMC1239896

[44] Ashburner, M. et al. Gene ontology: Tool for the unification of biology. *Nat. Genet.* **25**, 25–29 10.1038/75556 (2000). 10.1038/75556 PMC3037419 10802651

[45] Consortium, T. G. O. et al. The gene ontology knowledgebase in 2023. *Genetics* **224**, (2023). 10.1093/genetics/iyad031 PMC10158837 36866529

[46] Csardi, G. & Nepusz, T. The igraph software package for complex network research. *InterJournal Complex. Syst.*, 1695 (2006).

[47] Pedersen, T. L. & tidygraph: A Tidy API for Graph Manipulation. https://CRAN.R-project.org/package=tidygraph (2023).

[48] Street, K. et al. Slingshot: cell lineage and pseudotime inference for single-cell transcriptomics. *BMC Genom.* **19**, 1–16 (2018). 10.1186/s12864-018-4772-0 PMC6007078 29914354

