# Supplementary material for: Spatial transcriptomics unveils estrogen-modulated immune responses and structural alterations in the ectocervical mucosa of depot medroxyprogesterone acetate users
Source: Sci Rep. 2025 Jan 6;15:1014. doi: 10.1038/s41598-024-83775-9 (PMC11704007; doi:10.1038/s41598-024-83775-9)
Supplement: Supplementary file 1 — Supplementary Material 1 [file 41598_2024_83775_MOESM1_ESM.pdf]

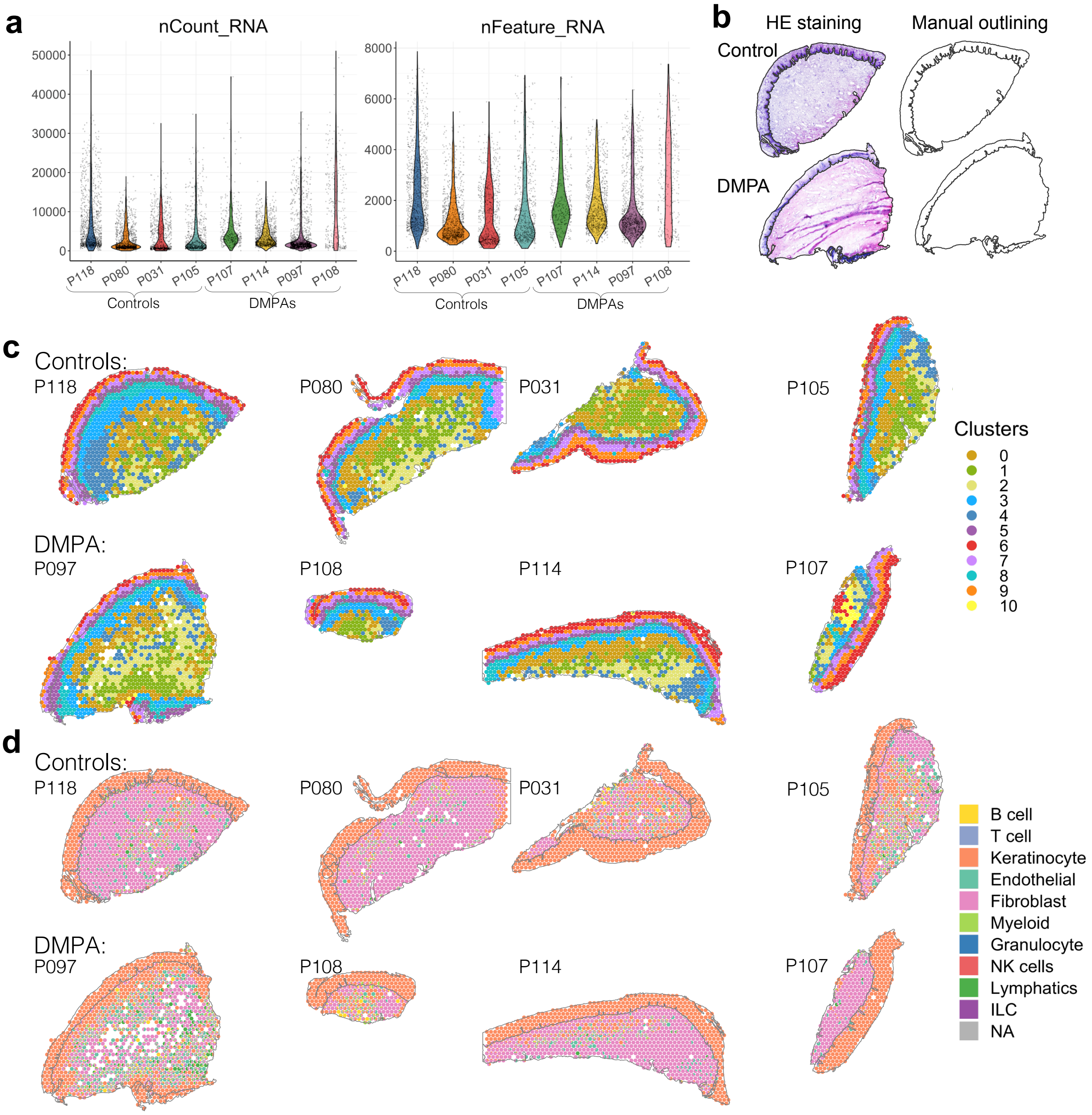

Supplementary Figure 1. Quality control, clustering, and deconvolution.

(**a**) Violin plots of total transcripts/counts and genes/ features for individual samples. (**b**) Manual outlining of the epithelium and submucosal regions. (**c**) Spots colored by clustering. (**d**) Spots colored by proportional estimates of cell populations.

a

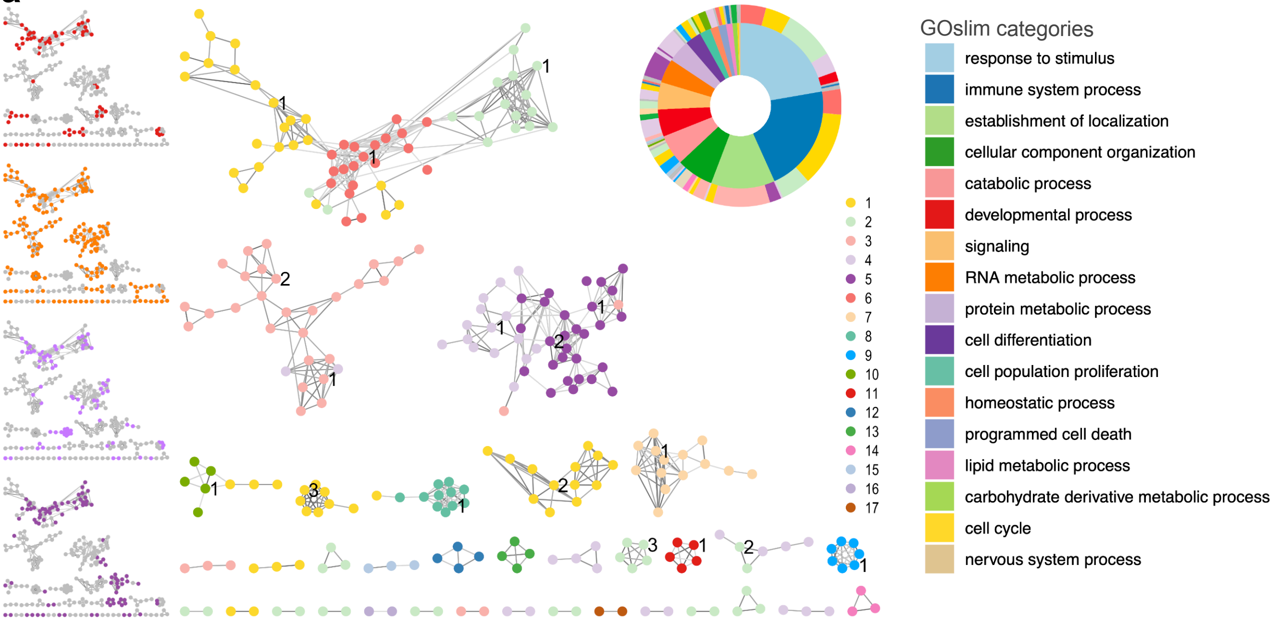

b

Cluster	pathway	Term	degree	ES
1^1	GO:0002449	lymphocyte mediated immunity	8	0.52
1^2	GO:0050863	regulation of T cell activation	8	0.45
1^3	GO:0002503	peptide antigen assembly with MHC class II protein complex	6	0.84
2^1	GO:0002253	activation of immune response	10	0.40
2^2	GO:0031349	positive regulation of defense response	3	0.44
2^3	GO:0001667	ameboidal-type cell migration	4	0.36
3^1	GO:0032386	regulation of intracellular transport	7	0.49
3^2	GO:0006626	protein targeting to mitochondrion	5	0.55
4^1	GO:0042176	regulation of protein catabolic process	7	0.46
5^1	GO:0006405	RNA export from nucleus	9	0.57
5^2	GO:0016071	mRNA metabolic process	15	0.44
6^1	GO:0002455	humoral immune response mediated by circulating immunoglobulin	18	0.78
7^1	GO:0009060	aerobic respiration	9	-0.43
8^1	GO:0043149	stress fiber assembly	9	0.53
9^1	GO:0031424	keratinization	6	-0.61
10^1	GO:0050678	regulation of epithelial cell proliferation	5	0.40
11^1	GO:0010273	detoxification of copper ion	4	-0.81

Supplementary Figure 2. Enrichment term clustering of epithelial GO terms.

(**a**) Graphical representation of enrichment results with nodes symbolizing terms, while edges connect terms that share genes within their respective gene sets. The smaller plots on the left show the significance of terms across the four distinct layers: superficial (red), upper intermediate (IM) (orange), lower IM (purple), and basal (dark purple). The pie chart indicates how the grouped terms relate to GOslim annotation. (**b**) The table includes the Enrichment Score (ES) and the number of connected nodes (degree) for selected terms marked in the graph with superscript numbers in the enrichment term cluster column.

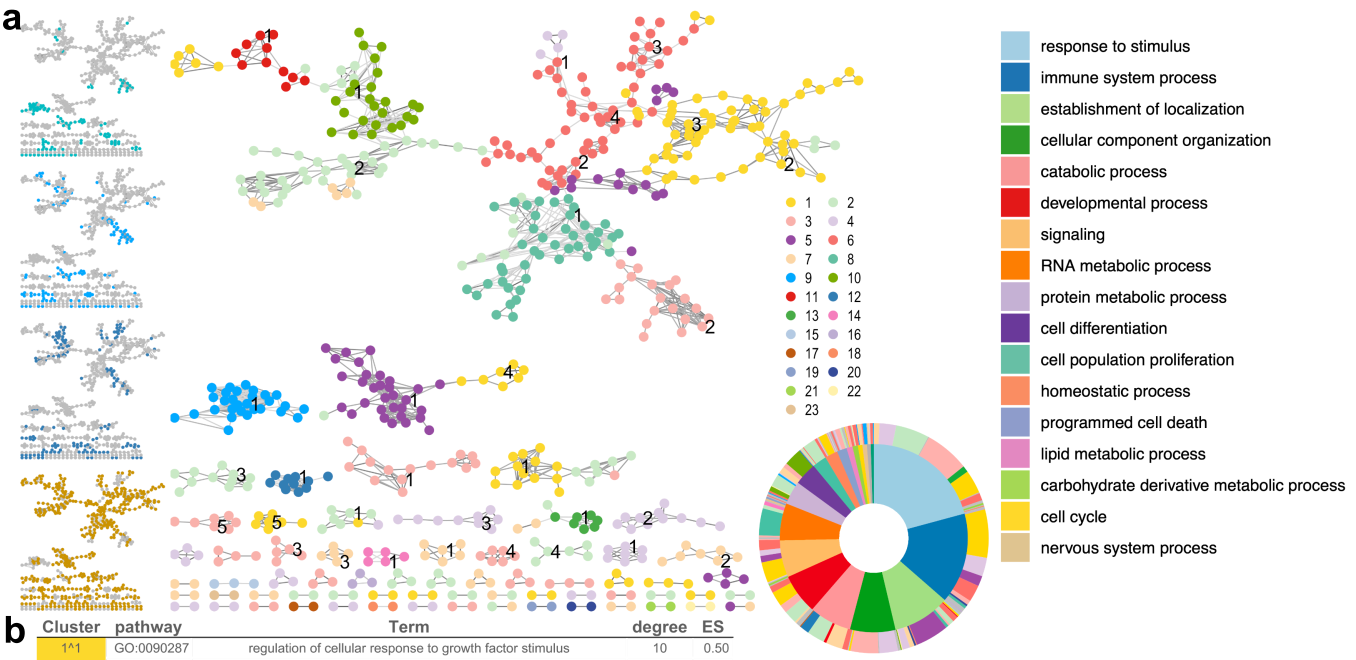

b

Cluster	pathway	Term	degree	ES
1^1	GO:0090287	regulation of cellular response to growth factor stimulus	10	0.50
1^2	GO:0045595	regulation of cell differentiation	7	0.43
1^3	GO:0010595	positive regulation of endothelial cell migration	10	0.55
1^4	GO:0016049	cell growth	4	0.45
1^5	GO:0090090	negative regulation of canonical Wnt signaling pathway	5	0.52
2^1	GO:0050727	regulation of inflammatory response	5	0.48
2^2	GO:0042176	regulation of protein catabolic process	9	0.51
2^3	GO:2001233	regulation of apoptotic signaling pathway	6	0.46
2^4	GO:1905897	regulation of response to endoplasmic reticulum stress	3	0.53
3^1	GO:0019731	antibacterial humoral response	7	0.76
3^2	GO:0002831	regulation of response to biotic stimulus	10	0.40
3^3	GO:0060267	positive regulation of respiratory burst	4	0.95
3^4	GO:0071375	cellular response to peptide hormone stimulus	5	0.47
3^5	GO:0071383	cellular response to steroid hormone stimulus	5	0.48
4^1	GO:0002062	chondrocyte differentiation	5	-0.50
4^2	GO:0019883	antigen processing and presentation of endogenous antigen	6	0.71
4^3	GO:0002418	immune response to tumor cell	5	0.74
5^1	GO:0030832	regulation of actin filament length	17	0.53
6^1	GO:0043568	positive regulation of insulin-like growth factor receptor signaling pathway	7	0.86
6^2	GO:2000677	regulation of transcription regulatory region DNA binding	7	0.63
6^3	GO:1902037	negative regulation of hematopoietic stem cell differentiation	6	0.94
6^4	GO:0031946	regulation of glucocorticoid biosynthetic process	5	0.83
7^1	GO:1901873	regulation of post-translational protein modification	5	0.49
7^2	GO:0031400	negative regulation of protein modification process	3	0.44
7^3	GO:1903047	mitotic cell cycle process	4	0.38
8^1	GO:0033157	regulation of intracellular protein transport	11	0.46
9^1	GO:0009060	aerobic respiration	21	-0.52
10^1	GO:0022613	ribonucleoprotein complex biogenesis	14	0.49
11^1	GO:0019058	viral life cycle	6	0.52
12^1	GO:1901985	positive regulation of protein acetylation	9	0.74
13^1	GO:0098754	detoxification	6	-0.53
14^1	GO:0010970	transport along microtubule	4	0.48

Supplementary Figure 3. Enrichment term clustering of submucosal GO terms.

(**a**) Graphical representation of enrichment results, nodes symbolize terms, while edges connect terms that share genes within their respective gene sets. The smaller plots on the left show the significance of terms across the four distinct clusters: cluster 8 (turquoise), cluster 3 (light blue), cluster 4 (dark blue) and cluster 0 (mustard). The pie chart indicates how the grouped terms relate to GOslim annotation. (**b**) The table includes the Enrichment Score (ES) and the number of connected nodes (degree) for selected terms marked in the graph with superscript numbers in the cluster column.

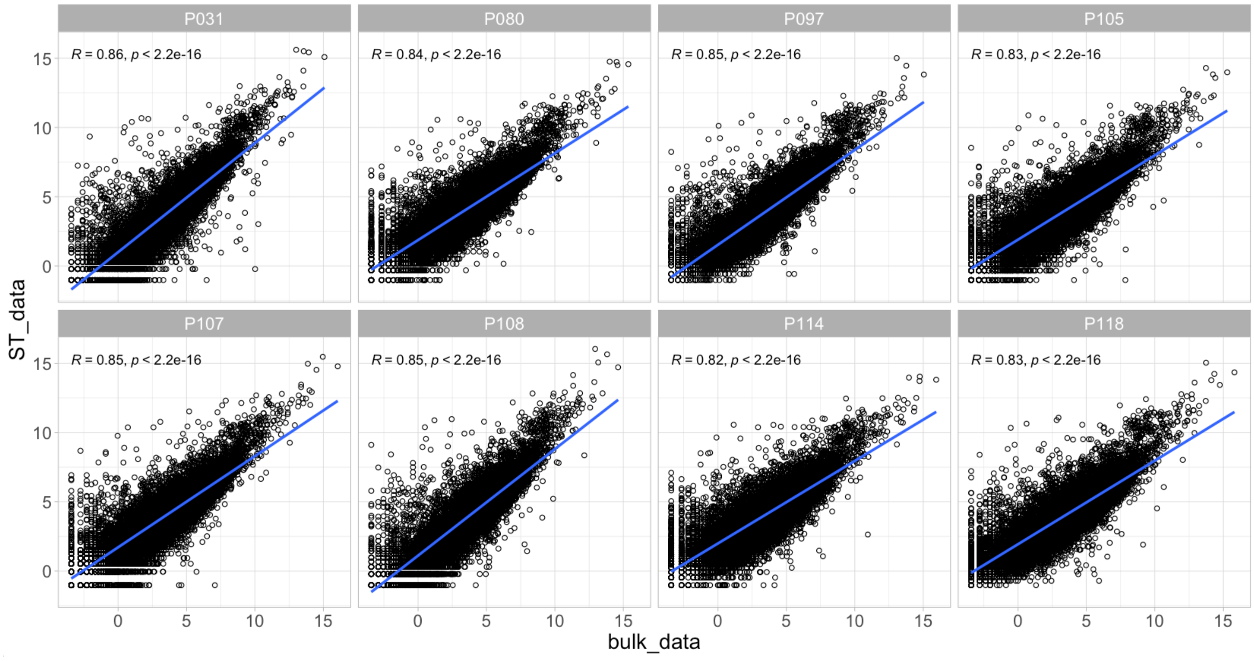

Supplementary Figure 4. Bulk data comparison.

Average gene expression values from each spatial transcriptomics sample plotted against the corresponding bulk data.
